# Effects of passive blood-flow-restriction and electromyostimulation on ICU-acquired weakness (ICUAW): a study protocol for a randomized-controlled, parallel-group clinical trial

**DOI:** 10.1186/s13063-025-08874-y

**Published:** 2025-06-01

**Authors:** Alexander Franz, Katharina Friederike Wolf, Julika Behrens, Björn Koos, Michael Adamzik, Stefan Felix Ehrentraut

**Affiliations:** 1 Department of Orthopedics and Trauma Surgery, University Hospital Bonn, Venusberg-Campus 1, Bonn, 53127 Germany; 2 Department of Trauma and Orthopedic Surgery, BG Klinik Ludwigshafen, Ludwigshafen, Germany; 3 Department of Anesthesiology and Intensive Care Medicine, University Hospital Bonn, Bonn, Germany; 4 Klinik für Anästhesiologie, Intensivmedizin und Schmerztherapie, Universitätsklinikum Knappschaftskrankenhaus Bochum, Bochum, Germany

**Keywords:** ICU-acquired weakness (ICUAW), Blood flow restriction (BFR), Electromyostimulation (EStim), Critical illness rehabilitation, Muscle atrophy prevention, Intensive care unit (ICU) interventions, Passive physiotherapy, Ischemic preconditioning (IPC), Critical care muscle metabolism, Randomized controlled trial (RCT)

## Abstract

**Background:**

Intensive care unit-acquired weakness (ICUAW) is a prevalent secondary disorder in critically ill patients, characterized by significant loss of muscle mass and strength, often leading to prolonged ICU stays, increased mortality, and reduced post-discharge quality of life. Despite guidelines recommending early mobilization, logistical challenges and inconclusive efficacy have limited its impact on ICUAW prevalence. This study aims to assess the feasibility, safety, and clinical efficacy of exclusively passive physiotherapeutic interventions, including blood flow restriction/ischemic preconditioning (BFR/IPC) and electromyostimulation (EStim), as potential alternatives for muscle preservation in ICU patients who are often sedated or unable to participate in active rehabilitation.

**Methods:**

This prospective, randomized controlled trial will recruit 120 patients from the surgical ICU at the University Hospital Bonn, who meet the inclusion criteria of a > 48-h ICU stay. Patients will be randomized into four groups: Sham-Control, BFR/IPC, EStim, and combined BFR/IPC + EStim. The study’s primary endpoints include feasibility and safety metrics, such as patient compliance and stress response, alongside secondary endpoints related to clinical outcomes like ICU length of stay, ICUAW prevalence, muscle mass preservation, and rehabilitation efficacy. Measurements include non-invasive assessments of muscle mass, intramuscular microdialysis to monitor metabolic and inflammatory markers, and health-related quality of life evaluations post-discharge.

**Discussion:**

Preliminary literature and a systematic review underscore the need for resource-efficient, non-invasive interventions in ICU settings. BFR/IPC and EStim present promising results, but existing data on their efficacy in ICU populations are limited. This study’s findings will provide foundational data on the viability of passive physiotherapy techniques in ICU settings, potentially improving patient outcomes and reducing healthcare costs associated with prolonged ICU stays. If successful, these results will inform a multicenter randomized trial to further evaluate these interventions. This research represents a crucial step in developing feasible rehabilitation protocols to mitigate ICUAW, addressing a critical gap in critical care management and rehabilitation.

**Trial registration:**

ClinicalTrials.gov DRKS00033592. Registered on March 05, 2024.

## Introduction

### Background and rationale {6a}

Intensive care unit acquired skeletal muscle weakness (ICU-acquired weakness, ICUAW) is a secondary disorder while patients are being treated for life-threatening conditions [1]. This syndrome includes critical illness polyneuropathy and myopathic changes in muscle function with associated significant loss of muscle mass (−17% in 10 days) and strength, and can be considered as a serious and expensive complication that compromises rehabilitation and full recovery [2]. In systemic reviews, ICUAW has a prevalence of approx. 43% and primarily affects the proximal limb and respiratory muscles [1]. The pathophysiology of this secondary condition is mostly unknown. Previous findings suggest a decrease in anabolic effector hormones with reduced protein biosynthesis due to immobility, mitochondrial dysfunction, and upregulated atrophy signaling with impaired autophagy processes [3]. Mitochondrial biogenesis is impaired during severe illness/sepsis [4, 5], and alterations between the interaction of the mitochondrial marker TFAM and TFMB2 have been shown to impact on survival [6].

Patients suffering from progressive muscle wasting show a longer ICU stay, higher re-intubation, and higher mortality rates before and after hospital discharge [7]. While the rate of ICU-survivors is steadily increasing due to modern drugs and interventions, ICUAW is causing an even higher number of physically severely impaired patients after discharge from the hospital. Epidemiological data from ICU survivors show a readmission rate of over 50% and mortality of 30%, especially during the first year after discharge [8]. If patients survive the first year, they suffer from significantly reduced physical capacity and limitations in their quality of life [9]. Furthermore, in patients of working age, a return-to-work rate of approximately 49% one year after discharge from the hospital is evident [8]. While the costs of an ICU stay with long-term intubation (> 20 days) and subsequent rehabilitation in the USA are estimated to be at around 300,000 $ [10], the estimated European post-discharge period with costs of around 30,000 € in the first and 18,000 € in the second year represents a major financial burden for the patient and the healthcare system as well [11].

#### Practical problems and potential passive solutions

Current guidelines for intensive care therapy recommend early mobilization of patients to prevent ICUAW. In clinical practice, however, physiotherapy exercises for ICU patients are extremely complex, only manageable with a considerable number of staff, and can rarely be carried out to the full extent, especially in sedated and intubated patients. An increasing shortage of personnel resources reduces the possibility of daily physiotherapy services as well. However, meta-analyses show that even early mobilization, if feasible, is not associated with a lower ICUAW prevalence or shorter ICU length of stay [12, 13]. These results could be due to two different reasons: (1) the interventions carried out were not intensive enough to provide the necessary stimulus to counteract muscle atrophy or (2) physical therapy treatment is unsuitable in the critical illness phase, as this is perceived as additional stress for the body or muscle preservation is physiologically not possible in this phase.

Therefore, the primary objective of this study is to investigate the clinical feasibility and safety of exclusively passive physiotherapeutic interventions in critical illness. The applied interventions, which consist of purely metabolic (blood flow restriction/ischemic preconditioning, BFR/IPC training), mechanical (electromyostimulation, EStim), or combined metabolic/mechanical load (BFR/IPC + EStim), will additionally be examined for their effects on the course of intensive therapy, physical and mental health/compliance of patients, and clinical outcomes (e.g., length of intensive care stay, length of hospital stay, prevalence of ICUAW, rehabilitation). It is intended that the results of this study will serve as a basis for subsequent projects regarding muscle metabolism and exercise therapy in ICUAW in a multi-center approach.

#### Level of evidence: narrative review

In preparation for this scientific study, an extensive literature analysis was carried out using the PRISMA guidelines in the following databases through December 2023: PubMed and Cochrane. The search about the impact of ICUAW was conducted using the following three searching combinations, with the database-specific boolean operators, were entered in each data base: (1) “intensive care” AND “muscle atrophy” (2) “intensive care” AND “ICUAW,” (3) “intensive care unit” AND “muscle loss” (4) “intensive care” AND “blood flow restriction training” AND (5) “intensive care” AND “electrical muscle stimulation”. The literature search included all randomized and non‐randomized studies, as well as systemic reviews and meta-analysis with adult patients up to the 31.12.2023. Articles in English and German were applied. In addition, citations of previously published meta-analyses and relevant reviews were manually searched, and the reference lists were reviewed to identify additional pertinent studies. The results of the five searching combinations were:

- *PubMed*: combination 1: 12 hits; combination 2: 21 hits; combination 3: 7 hits; combination 4: 0 hits; combination 4: 7 hits.
- *Cochrane*: combination 1: 88 hits; combination 2: 84 hits; combination 3: 28 hits; combination 4: 1 hits; combination 4: 46 hits.

#### Discussion of evidence

ICUAW shows a prevalence around 48% (95% CI, 39%, 56%) and is associated with an average loss of muscle mass of around 2.1%/day (M. rectus femoris: 95% CI, − 3.17, − 1.02) (level of evidence, LoE [14]: 1 A, 3.251 patients; [15]). Among these, older patients with pre- or sarcopenic conditions are particularly vulnerable to ICUAW and show higher ICU mortality, which is related to substantial loss of muscle mass (LoE: 1 A, 3.249 patients; [16]). The diagnosis of ICUAW is determined by sonographic muscle measurement in 85% of the studies included in meta-analyses (LoE: 1 A; [17]). The development of ICUAW is associated with a prolonged duration of mechanical ventilation, longer ICU and hospital stays, and mortality (LoE: 1B, 415 patients; [18]) and has a negative impact on rehabilitation after hospital discharge. Meta-analyses report increasing healthcare-related costs, 1-year mortality, and when it comes to survival after the ICU period, reduced physical activity outcomes compared to control groups over several years after hospital discharge (LoE: 1 A, 1.755 patients; [19]). These data can also be confirmed by observations from our research group that showed significantly reduced health-related quality of life in all tested domains, including mobility, life activities, and self-care. This was examined, using the WHO Disability Assessment Scale 2.0, 1 year after ICU discharge in patients who survived a long-term ICU-stay (unpublished data, data available for reviewers upon reasonable request).

Because of the reduced recovery of ICU survivors, the search for interventions to counteract the development of ICUAW and its associated complications is intensifying. The “early mobilization” (EM) recommended in the S3 guideline of the German Society for Anesthesiology and Intensive Care Medicine e.V. (DGAI) “Lagerungstherapie und Mobilisation von kritisch Erkrankten auf Intensivstationen” shows only inconclusive results in the meta-analyses (LoE 1 A, 1.304 patients; [12]) without showing a positive effect on the rehabilitation of patients after discharge from the ICU. There is evidence of short-term effects on physical-related outcomes (LoE: 1 A, 709 patients, [20]) if physiotherapeutic exercise is started within the first 48–72 h of intensive care (LoE: 1 A, 1.726 patients, [21]). The insufficient outcomes are mainly attributed to the heterogeneity of the patient population, their reduced trainability (no possibility of active participation), and the limited feasibility in the hospital, as physiotherapeutic exercise of intensive care patients is associated with high costs and personnel resources.

Therefore, new intervention methods are needed that can provide sufficient training against the development of ICUAW, which are safe for patients and require few costs and human resources. Within this regard, electrical muscle stimulation (EStim) appears to be a safe intervention strategy which can associated with EM have positive impact on extubation success in ICU patients (LoE: 1 A, 1.312 patients, [22]). However, the quality of evidence for EStim to reduce ICUAW and improve rehabilitation after ICU discharge is low, mainly due to bias of the RCT trials and the heterogeneity of the enrolled population (LoE: 1 A, 140 patients; [23]).

The blood flow restriction (BFR) technique was only applied in one RCT so far (*n* = 20) and was able to show a reduction in muscle atrophy (*P* = 0.001) without indicating adverse effects (LoE 1B; [24]). However, this study shows methodological limitations compared to our planned study: The study was conducted with standard inflatable cuffs, which are not suitable for BFR training [25]. For this reason and to increase patient safety, a CE-certified surgical-grade tourniquet autoregulation device (personalized BFR, Delfi Medical Inc., Vancouver, Canada) is used in our study protocol for the BFR/IPC exercise. The present study is the first to investigate the connection between metabolic (BFR/IPC) and mechanical (EStim) load and its influence on intensive therapy. Furthermore, the effect of the exercise on the muscle is to be described directly and validly on the basis of intramuscular measurements by microdialysis.

### Objectives {7}

ICUAW is a prevalent and severe complication in ICU patients, leading to significant muscle loss, prolonged hospital stays, and increased mortality. Despite the recommendation for early mobilization, existing interventions often fail to prevent or mitigate ICUAW due to practical constraints and insufficient intensity. This feasibility study aims to explore passive physiotherapeutic interventions—blood flow restriction/ischemic preconditioning (BFR/IPC) training, and electromyostimulation (EStim)—as alternative approaches which can be applied to all ICU patients, including those who are sedated or intubated. These techniques could circumvent the challenges of active physiotherapy and address muscle atrophy without exacerbating the patient’s condition or requiring extensive resources. By evaluating the safety, practicality, and effectiveness of these methods, the study seeks to address critical gaps in current ICU rehabilitation practices, potentially improving patient outcomes and reducing healthcare costs.

The present project aims to investigate these two questions by examining the effect of a purely metabolic (blood flow restriction/ischemic preconditioning (BFR/IPC) training), mechanical (electromyostimulation, EStim) or combined metabolic/mechanical load (BFR/IPC + EStim) on the physiology of the critically ill patient and clinical outcome (e.g., ICU length of stay, duration of hospitalization, prevalence of ICUAW, rehabilitation).

For this project, endpoints were defined that primarily reflect the clinical feasibility and safety of the intervention methods and their combinability with the clinical objective of muscle maintenance during ICU therapy and hospitalization. The following domains of outcomes include the evaluation of the impact of the interventions on the clinical rehabilitation process and the description of the underlying mechanisms of possible positive or negative effects on skeletal muscle physiology using invasive diagnostic monitoring.

#### Clinical feasibility, safety and acute muscle condition

To assess the clinical feasibility and safety of the applied interventions and diagnostic techniques on patients’ mental and physical health, the following endpoints are investigated in this study:

- *Feasibility*: Questionnaires regarding patient compliance (e.g., acute measure of recovery and stress, AEB) and possible stress response to the interventions (e.g., VAS 0–100 mm).
- *Global Health & Safety*: Changes in drug application for supporting the cardiovascular system (e.g., noradrenaline), use of analgetic (e.g., fentanyl) and sedation drugs (e.g., propofol) and changes in respiratory gases before, during and after each intervention session. Daily assessment of inflammation and coagulation parameters.

Since ICUAW and the resulting complications in the process of rehabilitation can be attributed to compromised muscle health, specific outcome parameters were selected that allow an objective description of possible positive or negative effects of the course of therapy, applied interventions, and further hospitalization on skeletal muscle conditions. These parameters will be partially recorded daily or before and after the intervention period, as well as by discharge from the ICU and hospital:

- *Muscle Health*: Muscle mass and fiber angle, muscle strength, neuromuscular activation, body composition (by bioelectrical impedance analysis).

#### Clinical endpoints

Since physical rehabilitation and recovery of functional resources after ICU hospitalization are associated with the preservation of muscle mass, the present project hypothesizes an impact of our passive training interventions on the clinical rehabilitation outcome. Therefore, the following endpoints will be recorded before and after the intervention period, by discharge from the ICU and hospital as well as during rehabilitation:

- *Hospitalization and mortality*: Length of ICU stay, length of mechanical ventilation, length of hospital stays, hospital readmission rate, hospital- and 28-day mortality.
- *Health-related quality of life and return to work*: WHODAS 2.0 on the day of discharge, 6 and 12 months after discharge, return to work rate, job level compared to previous status

#### Underlying mechanisms and physiological consequences by intensive care therapy and interventions

Given that the patient population included in this study can only be examined under certain conditions due to the state of sedation or mechanical ventilation, additional outcome parameters that can be collected independently of patient cooperation were selected. Furthermore, the selected endpoints make it possible to describe the internal load of the applied interventions, as well as to monitor the acute impact of the life-threatening disease and intensive therapy on changes in muscle metabolism and inflammation over the time of intervention:

- *Intramuscular measurements*: By implementing a microdialysis catheter (µDialysis, Stockholm, Sweden) into the *M. vastus lateralis*, which remains in the muscle for 8 days, it is possible to continuously measure metabolic impact (e.g., lactate, glucose, pyruvate) and inflammatory cytokines (e.g., IL-6, IL-8, IL-10, and TNF-α) of the ICU therapy and intervention on the muscle tissue. This minimally invasive technique enables the quantification of the acute influence of therapy on muscle tissue and to correlate it with changes in the other dimensions of the endpoints.
- *Systemic immune response*: For comparison between intramuscular immune responses to the disease, ICU therapy and the applied interventions, systemic immune response will be measured by analysis of inflammatory cytokines (TNFa, Interleukins) dependent on NFkB signaling [26] from the microdialysis samples and patient serum samples.

### Trial design {8}

**Fig. 1:**
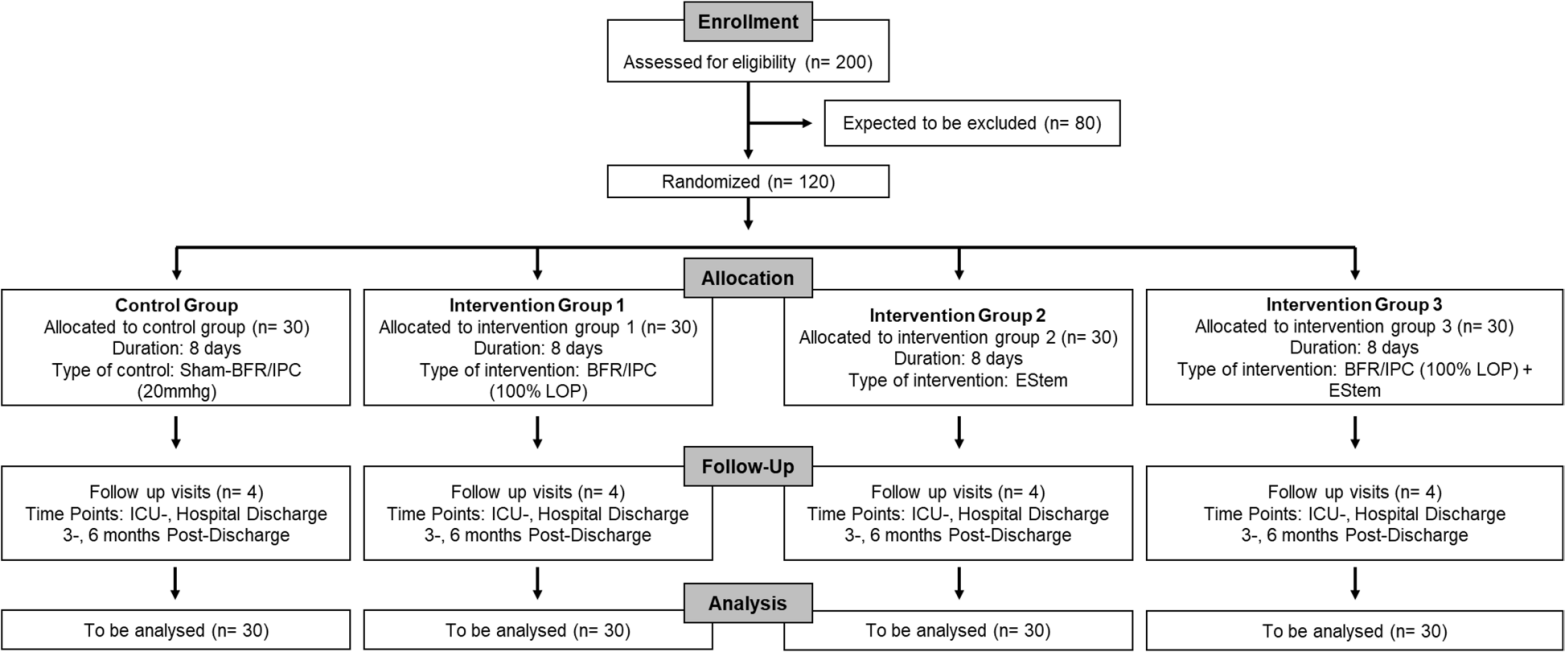
Trial design

This study is a prospective, randomized controlled trial within the clinical routine intensive care treatment of patients with a four parallel group study design (Fig. 1): (1) Sham-Control Group (Sham-BFR/IPC); (2) blood flow restriction/ischemic preconditioning (BFR/IPC) training; (3) electromyostimulation (EStim), and (4) BFR/IPC-Training + EStim.

It aims to investigate the feasibility, safety, and potential efficacy of passive physiotherapeutic interventions—blood flow restriction/ischemic preconditioning (BFR/IPC), electromyostimulation (EStim), and a combination of both—in patients with ICU-acquired weakness (ICUAW).

The primary hypothesis is:

Alternative hypothesis (H₁): Passive physiotherapeutic interventions (BFR/IPC, EStim, or both combined) are feasible and safe in ICU patients and lead to a measurable improvement in clinical and muscle-related outcomes compared to the sham control.

Null hypothesis (H₀): There is no difference in feasibility, safety, or clinical/muscle-related outcomes between passive physiotherapeutic interventions and the sham control.

The trial is not powered for definitive efficacy conclusions but will provide estimates and trends to inform future multicenter trials.

## Methods: participants, interventions, and outcomes

### Study setting {9}

The study protocol received approval from the local Ethics Committee of the University Hospital Bonn (Approval No.: 390/23) and is registered at the Clinical Trial Registry (DRKS00033592). The study will be performed at the intensive care unit (ICU) of the University Hospital Bonn (Bonn, Germany) as a cooperation between the Department of Anesthesia and Intensive Care and Department of Orthopedic and Trauma Surgery. All investigations and exercise sessions will be performed in the University Hospital Bonn.

### Eligibility criteria {10}

Patients will be recruited on the ICU of the University Hospital Bonn. The patient inclusion criteria are as follows: (1) The study includes all patients under intensive care monitoring and therapy with a length of stay > 48 h in the surgical intensive care units of the University Hospital Bonn. (2) All participating patients have signed the patient consent form for study participation as well as the privacy policy after comprehensive explanation, explanation and information or have been represented by the spouse as part of the spouse’s emergency representation after comprehensive explanation. (3) Patients aged > 18 years are included. Exclusion criteria for the study are: (1) acute or chronic infections of the extremities, (2) open wounds on the lower extremity, (3) Pregnancy and breastfeeding, (4) sickle cell anemia, and (5) former surgical interventions on the thigh vessels (e.g., stenting, bypassing).

### Who will take informed consent? {26a}

The informed consent will be taken by the clinical supervisors of the study.

If patients are conscious and capable of giving consent, they will be informed in detail about the study aims, procedures, risks, and benefits, and written informed consent will be obtained directly.

In cases where the patient is temporarily incapable of giving informed consent due to sedation or medical condition, legal representatives (court-appointed guardians or next of kin) will be contacted to obtain proxy consent.

Specifically, in accordance with current German legislation, the so-called “Ehegattennotvertretungsrecht” (spousal emergency representation) is applied if applicable. This allows the patient’s spouse to provide proxy consent in emergency situations, as confirmed by the local ethics committee.

Once patients regain full decision-making capacity, they will be re-informed, and consent will be reaffirmed or withdrawn as appropriate.

### Additional consent provisions for collection and use of participant data and biological specimens {26b}

No additional use of the data or biological samples beyond the scope of this study is planned. Accordingly, no specific consent for ancillary studies is required or obtained.

## Interventions

### Explanation for the choice of comparators {6b}

#### Sham-Control Group (Sham-BFR)

The control group is receiving the standard intensive care which also includes a daily physiotherapy session. To reduce the intervention bias, a daily"Sham"BFR intervention is also provided. The"Sham-BFR"intervention is performed in an intermittent manner on both thighs, according to the protocol of intervention group 1 (see below), but with the minimum standard pressure of 20 mmHg. This group serves to demonstrate the"clinically standard"progression of our test subject population and the changes are used as a basis for comparison with our intervention groups.

### Intervention description {11a}

#### Experimental intervention group 1: blood flow restriction/ischemic preconditioning (BFR/IPC) training

The first intervention group receives a daily BFR/IPC intervention of the lower extremities with 100% of the individual limb occlusion pressure (LOP). BFR training involves the application of tourniquets during a passive or active exercise intervention in order to provide an additive metabolic stimulus. The tourniquets are applied as proximal as possible to the thigh and a pressure is applied that temporarily occlude venous return with associated reductions/ complete blocking of arterial inflow [27]. For BFR/IPC training, a CE-certified surgical-grade tourniquet autoregulation device (CE-9–2200-001BFR-C, Delfi Medical Innovations Inc., Vancouver, Canada) is used. The applied exercise pressure is calculated by the individual limb occlusion pressure (LOP) using the BFR/IPC device at the start of each training session. The patient lies in bed at rest while the cuff is inflated until no more blood flow can be detected in the limb (= LOP). From this occlusion pressure, 100% is subsequently applied for the BFR/IPC intervention. The intervention protocol consists of daily exercise sessions of 50 min at intervals, whereby both thighs are exercised alternately (5 min occlusion + 5 min free flow, 5 times in total) over a period of 7 days [28].

#### Experimental intervention group 2: electromyostimulation (EStim)

The second intervention group receives a daily Sham-BFR/IPC intervention of 20 mmHg combined with electrical muscle stimulation training (EStim). For EStim, two self-adhesive electrodes (Axion Medical, Axion GMBH, Villengen-Schwennigen, Germany) are applied to the vastus lateralis muscle and two electrodes to the vastus medialis muscle. The stimulation parameters are as follows: frequency 50 Hz, work cycle 10 s stimulation followed by a 10-s pause and pulse width 400 µ. The EStim protocol is only applied during the occlusion phases of the Sham-BFR/IPC protocol. The intervention protocol consists of daily exercise sessions of 50 min at intervals, whereby both thighs are exercised alternately (5 min occlusion + 5 min free flow, 5 times in total) with the “Sham-BFR/IPC” pressure of 20 mmHg and the additional EStim Protocol over a period of 7 days. There is a coupling between the BFR and the EStim device (Mi-Theta 600; Cefar Compex), which only enables EStim during the phases of occlusion. If the limb is reperfused by releasing the air from the cuff, the EStim signal is interrupted and only reactivated when occlusion resumes.

#### Experimental intervention group 3: BFR/IPC-Training + EStim

The third intervention group combines BFR/IPC training with 100% of the individual limb occlusion pressure with EStim training. Within this intervention group both previously described interventions will be combined. For BFR/IPC training, 100% of the individual LOP is applied, whereas the EStim parameters are as follows: frequency 50 Hz, work cycle 10 s stimulation followed by a 10-s pause and pulse width 400 µ. The intervention protocol consists of daily exercise sessions of 50 min at intervals, whereby both thighs are exercised alternately (5 min occlusion + 5 min free flow, 5 times in total) over a period of 7 days. There is a coupling between the BFR/IPC and the EStim device (Mi-Theta 600; Cefar Compex), which only enables EStim during the phases of occlusion. If the limb is reperfused by releasing the air from the cuff, the EStim signal is interrupted and only reactivated when occlusion resumes.

### Criteria for discontinuing or modifying allocated interventions {11b}

Drop-out of a patient from the study: One or more of the following circumstances may lead to a patient discontinuing the study (these patients are counted as drop-outs): Withdrawal of consent by the patients or their legal representatives without giving reasons; Occurrence of an exclusion criterion; Violation of the study protocol.

### Strategies to improve adherence to interventions {11c}

#### Compliance to the intervention techniques

The compliance to the applied intervention techniques is assumed to be high based on experience from in-house studies in orthopedic patient populations. Passive BFR training was able to significantly reduce the subjective perception of pain and preserve muscle mass in the hospitalized phase after primary knee joint arthroplasty or spinal surgery (*unpublished data, data available for reviewers’ eyes only upon reasonable request*). Thereby, the technique was applied starting from the first day after surgery.

#### Drop outs and rate of loss to follow-up

Data from patients who withdraw their consent to participate during the intervention period of the study will remain in the analysis. If the patients consent to participate in the follow-ups, they will be analyzed as “intent-to-treat” within their original treatment group allocated by randomization.

Based on our experiences with the SepsisDataNet.NRW cohort (a large multicenter study on sepsis patients), we expect a loss to follow-up (30-day survival) of about 10%. Based on our experience from the health-related quality of life study in ECMO patients (see unpublished data above), we expect a follow-up rate of 75% from patients still alive at the time-point of 6-month follow-up. Due to the expected heterogeneity of the study population, no estimation of mortality in the study group can be done at this point.

### Relevant concomitant care permitted or prohibited during the trial {11 d}

#### Additional treatments

The present study will be carried out in addition to the routine treatment of the ICU to the existing guidelines of the S3 guideline of the German Society for Anesthesiology and Intensive Care Medicine e.V. (DGAI) “Lagerungstherapie und Mobilisation von kritisch Erkrankten auf Intensivstationen.” By participating in the study, patients do not receive any additional study-related procedures/medications except for the investigated study interventions. Furthermore, patients participating in this study will not be refused any intervention/medication if this is necessary in the course of their care.

### Provisions for post-trial care {30}

N/A

### Outcomes {12}

**Fig. 2:**
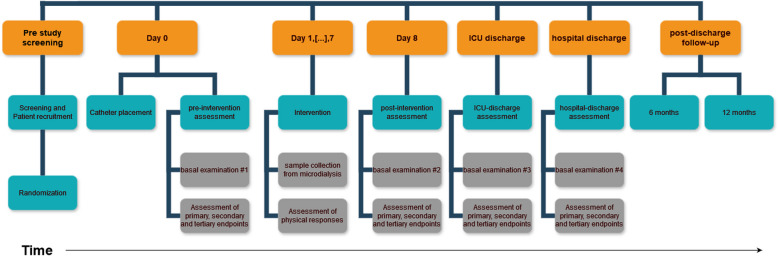
Study flow chart

For this research project, examinations will be divided into basal-, continuous-, and in-exercise measurement batteries (Fig. 2).

1. Basal examinations

All participating patients will go through a series of measurements before and after the intervention period of seven continuous days, as well as after discharge of the ICU and discharge of the hospital.

- Muscle Analysis I (Non-invasive):
  Muscle mass of the thigh muscles, which represents a correlation to the full body muscle mass [29], and fiber angle will be obtained by ultrasound and analyzed through a software-based post-processing pipeline. To determine grip strength, which shows a positive correlation with total body strength [30], a measurement of both hands is carried out using a hand dynamometer (Dynamo, Vald, Brisbane, Australia). An isometric maximum strength test is performed on both lower extremities during the following exercises: Dorsiflexion of the foot, plantar flexion of the foot, hip flexion, and knee extension. During this process, surface electromyography (Picoblue, Cometa, Bareggio, Italy) is used to analyze neuromuscular activation of the M. rectus femoris and M. vastus lateralis during the exercise.
- Global Health:
  For analyzing the global health status of the participating patients, routine laboratory measurements, containing, e.g., inflammation parameters (e.g., C-reactive protein, leucocytes), electrolyte concentrations, parameters of kidney- and liver function, will be measured daily. Additionally, the application of drugs for supporting the cardiovascular system (e.g., noradrenaline), analgesics (e.g., fentanyl), sedation (e.g., propofol), and antibiotics is monitored throughout the 7-day intervention phase as well as directly before and after each intervention training.
- Hospital length of stay, status at hospital discharge, economic effects:
  To assess the economic outcomes, data from the §21 Krankenhausentgeltgesetz data will be analyzed to measure and compare length of stay, status at hospital discharge and treatment costs between study groups
- Health-related Quality of Life:
  Patients are given questionnaires to record their health-related quality of life (Quality of Life, SF36; WHODAS 2.0) [31]. In addition, patients are asked to indicate their current pain perception on a 100-mm VAS.

(2) Continuous examinations

- Muscle Analysis II (Intramuscular, continuous measurements)
  By implementation of a microdialysis catheter into the M. vastus lateralis before the first basal examination is applied, it is possible to measure metabolic impact (e.g., lactate, glucose, pyruvate) and inflammatory cytokines (e.g., IL-6, IL-8, IL-10, and TNF-α) of the disease, ICU therapy, and study intervention on the muscle tissue continuously. This minimal-invasive technique enables the quantification of the acute influence of the therapy on the muscle tissue and to correlate it with changes in the other dimensions of the endpoints. The catheters are placed under sterile conditions and controlled by ultrasound after local anesthesia. In total, the catheter will stay in the muscle for 8 days. After implementation of the catheter, the flow rate at which the catheter is being flushed with the perfusion solution (NaCl) is set by a pump. During the day, the flow rate will be timed to 1 µl/min to obtain a sample of 180 µl every 3 h. Subsequent analyzation will be performed by ISCUSflex Microdialysis Analyzer (µDialysis, Stockholm, Sweden) and our cooperation partners at the Universitätsklinikum Knappschaftskrankenhaus Bochum, Ruhr-University Bochum.
- Systemic Immune Response
  In addition to intramuscular measurements to analyze immune function and responses in the skeletal muscle tissue, we will evaluate the systemic immune response of the intervention. EDTA blood samples will be obtained daily during the intervention period. EDTA blood is drawn and PBMCs are isolated using Ficoll gradient centrifugation. PBMCs are then spun unto a microscope slide (Cytospin) and fixed using formaldehyde solution. Mitochondrial protein interaction for TFAM and TFB2M will be analyzed using proximity ligation assay, as previously described [6]. Briefly, cells are permeabilized and blocked using Intercept TBS Blocking buffer (Licor) and incubated with both primary antibodies overnight. After washing cells are incubated with proximity probes against mouse and goat IgG. Oligonucleotides forming the DNA circle are hybridized and ligated and subsequently amplified using a compaction oligonucleotide (PMID 26202090). PLA signals are detected with fluorescent oligonucleotides and then visualized with a fluorescent microscope.
  In addition, ATP content of PBMCs is analyzed using the CellTiterGlo Kit (Promega). ROS induction is measured in plasma samples, which is isolated from EDTA blood. ROS is measured using CM-H2DCFDA a fluorescent probe that changes fluorescent spectrum upon oxidation by ROS. In addition, we will measure the cytokine response in serum samples of the patient. Specifically, we will measure the cytokines IL-6, IL-8, IL-10, and TNF-α which are known for their potential role in muscle injury.

(3) In-exercise measurements

- Physical and Mental Responses to Interventions:
  In order to analyze the impact of the interventions on patients mental and physical condition, before, each 10 min of exercise and after exercise, vital parameters such as blood pressure, pulse and oxygen saturation are measured. Furthermore, each from the beginning of the exercise intervention, each 10 min the applied medications for cardiovascular support, pain relief, and sedation are determined. For assessing the compliance of the patients to the interventions, subjective pain and exercise intensity will be obtained by visual analog scale (0–100 mm) and acute measure of recovery and stress (Akutmaß, Kurzskala, [32]).
- Blood Gas Analysis
  Before and after the exercise, a capillary blood sample is taken to visualize the venous blood gases (vBGA). The vBGA is used to determine carbon dioxide partial pressure (pCO_2_), oxygen partial pressure (pO_2_), oxygen content (ctO_2_), oxygen saturation (sO_2_), oxyhemoglobin fraction (FO_2_Hb), hemoglobin content (ctHb), oxygen half-saturation pressure (p50) and the concentration of lactate ([La^−^]), potassium ([K^+^]), sodium ([Na^+^]), calcium ([Ca^2+^]), bicarbonate ([HCO_3_^−^]), and chloride ([Cl^−^]).
- Intramuscular Monitoring
  During training interventions, the flow rate of the microdialysis catheter will be increased to 2 µl/min to get each 10 min a volume of 20 µl for analyzing the acute effects of the training interventions on muscle metabolism.

All outcome measures, there metric and timepoints are listed in Supplementary Table 2.

### Sample size {14}

**Fig. 3:**
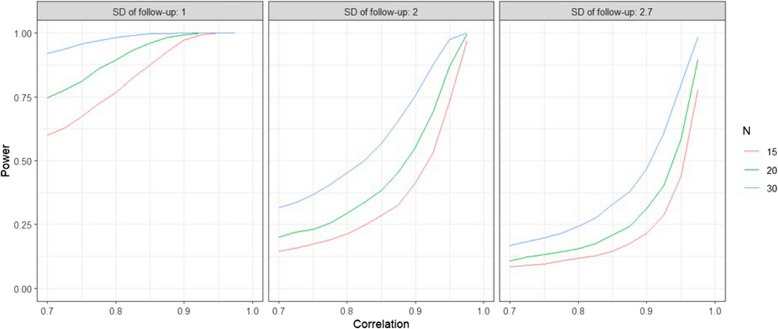
Simulation results for the arm-specific power of an ANCOVA with Dunnett test based on 10.000 simulations

In a previous study [24] in intensive care unit patients with muscle thickness as outcome, a baseline value of 11.2 mm (standard deviation (SD) of 2.7 mm) was observed with a reduction of 2.8 mm (SD 0.7 mm) in the control group vs. 2.1 mm (SD 0.9 mm) in the blood flow restriction group, with a sample size of 20 patients in each group. Using this as assumption in the sample size calculation, we set up a simulation study with R (version 4.2.2) to investigate the statistical power of an ANCOVA with Dunnett’s test to compare each of the three treatment arms to the control group. We assumed various scenarios for the standard deviation of the follow-up measurement from small (1 mm) over moderate (2 mm) to large (2.7 mm) as well as conservative to optimistic values for the correlation between baseline and follow-up measurement (rho between 0.7 and 1) (Fig. 3).

The simulation results indicate that under the assumption of a moderate standard deviation and relatively large correlation (rho > 0.9), a sample size of *N* = 30 per arm provides a statistical power of at least 75% for each arm, i.e., the marginal power. We view this as sufficient statistical power to explore the effectiveness of the different interventions for the first time under relatively conservative, and to be able to identify a promising treatment to be evaluated in a future trial.

### Recruitment {15}

The University Hospital Bonn has one of the largest surgical intensive care capacities in Germany (88 surgical intensive care beds). In the course of the feasibility study, all patients who meet the inclusion criteria can be enrolled in this study. To extend the inclusion to patients under sedation or mechanical ventilation, the consent to participate in the study by the “Ehegattennotvertretungsrecht” was also added and ethically confirmed. This allows the patient’s legal spouse, if they are not currently able to give consent, to be contacted and informed, and to confirm or decline participation in the study. During the pre-testing phase, a participation rate of 85% was achieved.

The feasibility of recruitment is derived from our experiences with the recruitment in previous intensive care projects. Approximately 3500 cases are treated at our institution’s surgical ICUs annually. Of these, approximately 5–10% have an extended stay < 7 days, making them good candidates for the proposed study. At the current rate of extended stay ICU patients, we expect to screen 25 patients per month. Of these we plan to recruit approximately 5–6 patients per month into the project, which means that in 36 months we can recruit up to 180 patients.

## Assignment of interventions: allocation

### Sequence generation {16a}

All patients included in the study will be randomized in one of our four study groups. Randomization is performed using a computer-generated block randomization list [33], using the statistical software R and the package"blockrand". The allocation is carried out according to the randomization list in numerical order of the pseudonyms. In order to obtain a balanced study design, two lists (pre- and post-interim analysis) will be created, each containing 30 patients per study arm. To avoid a bias by the participants, we have included a “Sham-Control” group which is receiving a “Sham-BFR/IPC” intervention with a fixed pressure of 20 mmHg to reduce the intervention bias. This also gives the patients in the control group the experience of taking part in an intervention.

#### Criteria for discontinuing or modifying allocated interventions

Drop-out of a patient from the study: One or more of the following circumstances may lead to a patient discontinuing the study (these patients are counted as drop-outs): Withdrawal of consent by the patients or their legal representatives without giving reasons; Occurrence of an exclusion criterion; Violation of the study protocol.

### Concealment mechanism {16b}

All potential participants will initially be screened for the eligibility criteria via physical examination on the ICU. Subsequently, all individuals who pass the initial screening and are interested in the study will sign a written informed consent and complete the baseline assessment. Following the baseline assessment, participants will be randomly allocated to one of the four study groups.

### Implementation {16c}

The results of the block randomization are printed out and distributed among 120 identical-looking, sealed letters numbered in ascending order from 1 to 120. The research assistants responsible for enrolment were not informed about the randomization list and accept the letters in order.

## Assignment of interventions: blinding

### Who will be blinded {17a}

Although full blinding of participants is not feasible due to the perceptible nature of the interventions (e.g., tourniquet pressure, electrical stimulation), a sham intervention protocol is employed to reduce performance and expectation bias.

Specifically, the control group receives a daily “Sham-BFR/IPC” procedure using a low, non-therapeutic pressure (20 mmHg), mimicking the setup and procedure of the BFR/IPC group without providing physiological stimulus. This approach ensures that participants experience a similar intervention environment and procedure across all groups, regardless of group allocation.

While participants may still perceive differences (particularly if conscious), the standardized application of equipment and procedures helps mitigate subjective bias. Outcome assessors and data analysts remain fully blinded to group assignments.

### Procedure for unblinding if needed {17b}

In this study, only outcome assessors are blinded to group allocation. Participants and clinical staff are not blinded due to the perceptible nature of the interventions.

Unblinding of outcome assessors will only take place if required for participant safety or in exceptional circumstances affecting data validity. Any such unblinding will be initiated and documented by the principal investigators.

Data analysts will receive only anonymized and already unblinded datasets after completion of data collection. Since no interim analyses are planned, unblinding of data analysts during the trial is neither necessary nor possible.

## Data collection and management

### Plans for assessment and collection of outcomes {18a}

#### Validity of the applied measuring tools

The measurement tools employed in this study have demonstrated strong validity and reliability in assessing muscle thickness, health-related quality of life, and pain intensity. Ultrasound imaging is recognized as a reliable method for measuring muscle thickness, with studies indicating high intra-rater reliability (ICC = 0.985–0.998) and inter-rater reliability (ICC = 0.868–0.964), as well as significant correlations with magnetic resonance imaging measurements (*r* = 0.789, *p* < 0.001) [34]. The World Health Organization Disability Assessment Schedule 2.0 (WHODAS 2.0) has been validated as a reliable instrument for evaluating health-related quality of life, demonstrating excellent internal consistency (Cronbach’s *α* = 0.91) and moderate correlations with established measures such as the EuroQoL visual analog scale (*r* = − 0.72, *p* < 0.001) [35]. The visual analog scale (VAS) is widely utilized for pain assessment and has been validated in various populations, showing high sensitivity and reliability in capturing subjective pain intensity. Collectively, these tools provide a comprehensive and valid assessment of the outcomes related to the myostimulation protocol.

#### Data acquisition

All patients will undergo the standardized study protocol. Before the start of the project, research assistants will be trained to standardized protocols to obtain the outcome parameter. Per each day, each measurement will be repeated thrice per time point. The median of these technical replicates will be used as the repeated measurement value for said time point.

All outcome data will be collected using standardized data collection forms. These forms are paper-based and will be completed manually by trained research staff during or immediately after each measurement or intervention session.

The collected data will then be entered into a secure, password-protected instance of REDCap (Research Electronic Data Capture), hosted at the University Hospital Bonn. Access to REDCap is restricted to authorized study personnel.

To ensure data completeness and accuracy, the following quality assurance measures are in place:

- All study personnel are trained in data collection protocols before study initiation.
- Each measurement is performed three times, and the median is used for analysis.
- Data entry is performed by one investigator and checked by a second member of the research team (dual entry control).
- REDCap includes validation rules and logic checks to minimize entry errors.
- Monthly internal audits will be performed by the principal investigators to identify missing or inconsistent data.

All changes in the database are tracked automatically and time-stamped to ensure full auditability.

### Plans to promote participant retention and complete follow-up {18b}

Given that some of the patients will be coming from far away, follow-up after hospital discharge will be done via telephone calls (Quality-of-Life Assessment: WHODAS 2.0, Return-to-Work).

### Data management {19}

#### Data storage

The data is stored using Research Electronic Data Capture (REDCap). Data entry is managed by the principal investigators, which provides an additional check on the data completeness. Access is strictly restricted to authorized study personnel through personalized login credentials and two-factor authentication. During the trial, data will be stored *pseudonymized*, using a study ID code without direct identifiers. The linkage between personal identifiers and study IDs is stored separately on an encrypted, access-restricted device under the control of the principal investigators.

#### Data analysis

For each study arm and time point the pooled median values will be compared against the control group and the other respective study groups. The data analysis is carried out by a blinded biostatistician.

After trial completion and data analysis, the pseudonymization list will be destroyed, and data will be converted to fully anonymized form.

All anonymized data will be archived for 10 years in accordance with institutional and national data retention policies for clinical research. The data will be stored on institutional servers with secure backup and will be deleted irreversibly after the retention period.

### Confidentiality {27}

All data collected in the process of the study are protected by data protection laws. Personal data will not be disclosed or made accessible to the public by the principal investigators (Dr. med. Alexander Franz, PD Dr. med. Stefan Ehrentraut) or any other person involved in conducting the study. Disclosure and data storage will only take place with pseudonymized patient data. The pseudonymization is carried out by means of an ascending series of numbers by the principal investigators. Only the investigators may assign the personal data to the study data. The assignment code is stored on an external hard disk and is managed by the principal investigators. After completion of the study, the assignment code is deleted and the data management is changed from pseudonymization to anonymization. Data analysis will be performed after anonymization. All collected data collected will be stored for a period of 10 years and deleted afterwards.

### Plans for collection, laboratory evaluation, and storage of biological specimens for genetic or molecular analysis in this trial/future use {33}

#### Plans for collection, laboratory evaluation, and storage of biological samples for molecular analysis in this trial

### Molecular analysis

Continuous sample collection via the intramuscular microdialysis catheter is performed at a steady flow rate of 1 µl/min. This allows a sample volume of 180 µl to be collected every 3 h which are subsequently stored at 4 °C. The samples collected within 24 h are then pooled and divided into 250-µl aliquots and stored at − 80 °C. During training interventions, the flow rate of the microdialysis catheter will be increased to 2 µl/min to get each 10 min a volume of 20 µl for analyzing the acute effects of the training interventions on muscle metabolism.

Subsequent analyzation for metabolic parameters will be performed by enzymatic-colorimetric microdialysis analyzer (ISCUSflex Microdialysis Analyzer, µDialysis AB, Stockholm, Sweden) [36].

## Statistical methods

### Statistical methods for primary and secondary outcomes {20a}

#### Data acquisition

All patients will undergo the standardized study protocol. Per each day, measurements will be repeated thrice per time point. The median of these technical replicates will be used as the repeated measurement value for said time point. For descriptive purposes values will be reported as median ± interquartile range or absolute number and percent, where appropriate.

Primary analysis method will be an ANCOVA with Dunnett’s test with the measurement after 7 days as outcome and adjusted for the baseline value. Additionally, mixed models will be fitted to assess the sensitivity of the results under consideration of all measurement points. Health-related quality of life will be analyzed using mixed models for repeated measurements. Again, joint models may be considered to take a considerable proportion of missing data into account. Time-to-event outcomes, such as length of ICU stay, length of hospital stay, and time to return to work, will be analyzed using Kaplan–Meier estimates and Cox regression models.

All primary, secondary, and other exploratory outcomes will also be reported descriptively. Patients who decide to drop out of the study prior to completion of the intervention will remain in the study analysis and their respective study cohort following an intention-to-treat analysis. Recruitment will commence until the overall number of 120.

### Interim analyses {21b}

No interim analyses are planned for this trial. The full dataset will be analyzed only after completion of data collection for all participants. This decision was made due to the feasibility-focused design and the relatively small sample size, which would not justify interim statistical testing.

### Methods for additional analyses (e.g., subgroup analyses) {20b}

No predefined subgroup or additional exploratory analyses are planned at this stage.

Given the feasibility nature of this trial and the limited sample size, the study is not powered to detect differences across subgroups. Any future subgroup analysis will require confirmation in larger, follow-up trials.

### Methods in analysis to handle protocol non-adherence and any statistical methods to handle missing data {20c}

Multiple imputation will be used to take into account missing values, provided data is missing at random. If there is a considerable amount of missing data due to protocol violations or discontinuation, application of joint models will be considered to adequately take this information into account.

Data from patients who withdraw their consent to participate during the intervention period of the study will remain in the analysis. If the patients consent to participate in the follow-ups, they will be analyzed as “intent-to-treat” within their original treatment group allocated by randomization.

Based on our experiences with the SepsisDataNet.NRW cohort (a large multicenter study on sepsis patients), we expect a loss to follow-up (30-day survival) of about 10%. Based on our experience from the health-related quality of life study in ECMO patients (see unpublished data above), we expect a follow-up rate of 75% from patients still alive at the time-point of 6-month follow-up. Due to the expected heterogeneity of the study population, no estimation of mortality in the study group can be done at this point.

### Plans to give access to the full protocol, participant-leveldata and statistical code {31c}

All information about the study design and the collected, anonymized data of the study that are not used in the final publication can be provided upon request to the authors.

## Oversight and monitoring

### Composition of the coordinating center and trial steering committee {5d}

This is a single-center, investigator-initiated trial without a formal trial steering committee or coordinating center.

The trial is managed and overseen by the principal investigators (Dr. med. Alexander Franz and PD Dr. med. Stefan F. Ehrentraut), who are responsible for overall study coordination, protocol adherence, safety oversight, and communication with the ethics committee.

The day-to-day conduct of the trial, including patient recruitment, intervention delivery, and data collection, is managed by the research team from the Departments of Anesthesiology and Intensive Care Medicine and Orthopedics and Trauma Surgery at University Hospital Bonn.

Weekly internal meetings are held to monitor study progress, discuss any issues, and ensure compliance with study procedures and timelines.

### Composition of the data monitoring committee, its role and reporting structure {21a}

For this clinical study, monitoring is not being conducted due to its primary focus on evaluating the design, interventions, and methodology in a limited scope, providing a foundation for a future, larger multicenter trial. The procedures and interventions conducted in this study are safe, well-established, routinely used in comparable studies and following standardized protocols that minimize the risk of deviations. The research team has extensive experience in conducting clinical studies and is well-trained and familiar with the study’s interventions and safety measures. Additionally, the collection and management of study data comply with applicable data protection regulations. Data will be stored pseudonymously, analyzed anonymously, and securely maintained. Lastly, the study was approved by the clinical ethics committee of the University Hospital Bonn as outside the CTR and MDR guidelines, ensuring strict compliance with all ethical guidelines without the need for additional monitoring.

### Adverse event reporting and harms {22}

#### Adverse events and serious adverse events grading

## Adverse event grading

Adverse events will be graded using standard criteria. Relationship of event to the study procedure will be determined by the study physician.

- GRADE 1 (Mild) Transient or mild discomfort (< 48 h); no medical intervention/therapy required
- GRADE 2 (Moderate) Mild to moderate limitation in activity—some assistance may be needed; no or minimal medical intervention/therapy required
- GRADE 3 (Severe) Marked limitation in activity, some assistance usually required; medical intervention/therapy required, hospitalizations possible
- GRADE 4 (Life-threatening) Extreme limitation in activity, significant assistance required; significant medical intervention/therapy required, hospitalization or hospice care probable.

### Relationship assignment

The relationship of the adverse event to participation in the study will be assessed as either:

- Definitely related
- Probably related
- Possibly related
- Unlikely related
- Unrelated

#### Adverse event reporting procedures

- Reporting responsibilities:
  - *○ Participants*: Report any unusual or unexpected symptoms or experiences to study staff immediately by phone or by mobile app
  - *○ Study staff*: Document all reported events promptly and ensure proper communication with the principal investigator (PI).
- Identification and classification:
  - *○ Type of Event*: Describe the nature of the event (e.g., physical injury, psychological distress).
  - *○ Severity*: Grade the severity from Grade 1 to Grade 4.
  - *○ Causality assessment*: Determine whether the AE is related to the study intervention (unrelated, unlikely-, possibly-, probably-, or definitely related).
- Documentation:
  - ○ Record the AE in the *Adverse Event Log* or study-specific Case Report Form.
  - ○ Include the following details:
    1. Date and time of onset.
    2. Date and time of onset.
    3. Severity and duration.
    4. Actions taken (e.g., treatment provided, intervention discontinued).
    5. Outcome (resolved, ongoing, worsened).
- Immediate reporting of serious adverse events:
  Any serious adverse event (e.g., life-threatening reaction, hospitalization) must be reported to the ethics committee and study sponsor within 24 h. Include:
  - ○ Detailed description of the event.
  - ○ Detailed description of the event.
- Follow-up:
  - ○ Monitor the participant until the AE resolves or stabilizes.
  - ○ Update the AE report with follow-up information.
- Regulatory reporting:
  - ○ Submit regular AE summaries to the ethics committee per protocol requirements.
  - ○ Ensure timely reporting of unexpected or related SAEs to relevant authorities.
- Preventive actions:
  - ○ Review AEs regularly to identify trends or risk factors.
  - ○ Update the study protocol, participant materials, or safety measures if needed.

Given the nature of the interventions (blood flow restriction, electrical stimulation), potential expected adverse events include:

- Local skin irritation or redness at electrode or cuff sites
- Transient muscle discomfort or soreness
- Mild circulatory changes (e.g., temporary blood pressure fluctuations)

*All participants will be systematically monitored* for adverse events throughout the study, including vital signs before, during, and after each intervention. Clinical staff will record any observed events, and participants (or their representatives) will be asked about symptoms using *open-ended questions* as well as *standardized observation forms*.

Any *unexpected harms* will also be documented and reviewed by the principal investigators. All adverse events will be graded based on severity and relatedness to the study intervention using the predefined grading scale in the protocol (Grades 1–4).

While we do not use MedDRA coding, all events will be categorized using standardized medical terminology in accordance with ICH-GCP and institutional reporting standards.

*All adverse events of Grade 2 or higher*, or those classified as serious (SAEs), will be reported in trial publications. Grade 1 (mild) events will be summarized descriptively unless they are unusually frequent or clustered.

### Frequency and plans for auditing trial conduct {23}

No formal auditing of the trial conduct is planned.

This decision is based on the study’s single-center design, limited scope (feasibility and safety), and the nature of the interventions, which are non-/minimally invasive and routinely used in clinical practice.

The trial will be internally supervised by the principal investigators through regular team meetings and protocol compliance checks. All relevant procedures are performed according to standard operating procedures, and data handling follows institutional GCP-compliant practices.

### Plans for communicating important protocol amendments to relevant parties (e.g., trial participants, ethical committees) {25}

No protocol amendments are currently anticipated during the course of this study.

However, if changes become necessary (e.g., due to safety concerns, feasibility issues, or regulatory feedback), these amendments will be submitted for review and approval to the local ethics committee of the University Hospital Bonn before implementation.

All relevant parties, including clinical staff involved in the study and participants (or their legal representatives), will be informed of the nature and rationale of the changes in an appropriate and timely manner.

Any amendments will also be updated in the trial registry (DRKS00033592) and reported in any resulting publications.

This project was presented to ICU survivor from the Department of Anesthesiology and Surgical Intensive Care Medicine. The purpose of the study, interventions, and primary clinical endpoints were explained in detail and in layman’s terms. The patients had the opportunity to provide input on the trial procedures and investigation parameters.

After completion of the study, it is planned to organize a patient symposium at the University Hospital Bonn with public access in order to report comprehensively on the results and clinical significance of this study. In this regard, ICU survivors and participating patients of the study will be invited to provide feedback on their participation and input on the study. Subsequently, recommendations for possible modifications to the study design, interventions, or clinical outcomes will be used for subsequent multicenter studies on the impact of exercise therapy during critical illness.

### Dissemination plans {31a}

The findings of this study will be disseminated through multiple channels to ensure broad accessibility and impact. The results will be submitted for publication in a peer-reviewed scientific journal specializing in rehabilitation, neuromuscular physiology, or clinical interventions. Additionally, the study will be presented at relevant national and international conferences to facilitate discussion among researchers, clinicians, and policymakers. To enhance accessibility, key findings will be shared through institutional and professional networks, as well as on open-access research repositories when possible. Moreover, a summary of the results will be communicated to participants and stakeholders to ensure knowledge translation into clinical practice. Where appropriate, collaborations with healthcare organizations and patient advocacy groups will be sought to maximize the real-world application of the findings.

### Authorship {31b}

Authorship of future trial publications will follow the *ICMJE (International Committee of Medical Journal Editors)* guidelines.

Individuals will be eligible for authorship if they have made substantial contributions to:

- The conception or design of the study
- Acquisition, analysis or interpretation of data
- Drafting or critically revising the manuscript for important intellectual content.

All authors will approve the final manuscript and agree to be accountable for all aspects of the work.

No professional medical writers or editorial services have been or will be used in the preparation of this study protocol or any subsequent publications.

## Discussion

So far, there is a scarcity of data regarding the effect of different physical therapy approaches on global and muscular health during ICU stay. This is even more profound in regard to sedated and or ventilated long-term ICU patients. The current German guidelines recommend early mobilization for intensive care patients, even for those undergoing extracorporeal membrane oxygenation [12]. However, the level of evidence is low, especially when it comes to neuromuscular electrostimulation or passive interventions like BFR/IPC. Here, we will identify the feasibility of a 7-day intervention course in combination with different treatment modalities and their impact on muscle mass preservation, muscle metabolism, and patient reported outcomes (i.e., WHODAS 2.0). This will allow for identifying unintended outcomes and potential adverse effects of EStim and/or BFR/IPC treatment in critically ill patients. If successful, we will generate new evidence and knowledge to hopefully justify a multi-center randomized controlled trial.

## Trial status

In preparation.

## Data Availability

The datasets generated and/or analyzed during the current study are not publicly available due local data protection rules but are available from the corresponding author on reasonable request and evaluation of said request by the local data protection officer/board.

## Abbreviations

AEB: Acute measure of recovery and stress
ANCOVA: Analysis of covariance
ATP: Adenosine triphosphate
BFR: Blood flow restriction
BFR/IPC: Blood flow restriction/ischemic preconditioning
CI: Confidence interval
DGAI: German Society for Anesthesiology and Intensive Care Medicine e.V.
EDTA: Ethylenediaminetetraacetic acid
EStim: Electromyostimulation
FO2Hb: Oxyhemoglobin fraction
ICU: Intensive care unit
ICUAW: Intensive care unit-acquired weakness
IL-x: Interleukin-x
LoE: Level of evidence
LOP: Limb occlusion pressure
NFkB: Nuclear Factor kappa-B
p50: Oxygen Half-Saturation Pressure
PBMC: Peripheral blood mononuclear cell
PLA: Proximity ligation assay
PRISMA: Preferred Reporting Items for Systematic Reviews and Meta-Analyses
RCT: Randomized controlled trial
ROS: Reactive Oxygen Species
sO_2_: Oxygen saturation
TFAM: Transcription Factor A, Mitochondrial
TFB2M: Transcription Factor B2, Mitochondrial
TNF-α: Tumor Necrosis Factor Alpha
VAS: Visual analog scale
vBGA: Venous blood gas analysis
WHODAS: World Health Organization Disability Assessment Scale
